# Sex differences in normal fetal regional brain apparent diffusion coefficient changes assessed by *in utero* DWI

**DOI:** 10.3389/fped.2024.1354475

**Published:** 2024-03-19

**Authors:** Jing-Ya Ren, Ming Zhu, Su-Zhen Dong

**Affiliations:** Department of Radiology, Shanghai Children’s Medical Center, School of Medicine, Shanghai Jiao Tong University, Shanghai, China

**Keywords:** fetus, brain development, diffusion-weighted imaging, apparent diffusion coefficient, sex differences

## Abstract

**Objective:**

There are differences in the vulnerability of male and female fetal brains to adverse intrauterine exposure, preterm birth, and associated perinatal brain injury. The main objective of this study was to identify any statistically significant difference in the change of apparent diffusion coefficient (ADC) in the intracranial regions of male and female fetuses in the second and third trimesters.

**Methods:**

Diffusion-weighted imaging (DWI) was performed in 200 fetuses between 20 and 37 gestational ages (GA) with normal results or suspicious results on sonography followed by structural MRI. Pairwise ADC values of the regions of interest (ROIs) were manually delineated on either side of the cerebral white matter: frontal white matter (FWM), parietal white matter (PWM), occipital white matter (OWM), temporal white matter (TWM), basal ganglia (BG), thalamus (THA), cerebellar hemisphere (CBM), and a single measurement in the pons. The changes in these values were studied over the gestational range, along with potential sex differences and asymmetries of the cerebral hemispheres.

**Results:**

During the third trimester, ADC values in OWM, TWM, and CBM were significantly higher in male fetuses than those in female fetuses (*p *< 0.05). After the correction of false-discovery rates (FDR), the difference in CBM was the only statistically significant (*p =* 0.0032). However, the decreased rate of ADC values in male fetuses in CWM (except for FWM), BG, THA, CBM, and pons was higher than that in female fetuses during the second and third trimesters.

**Conclusions:**

We have shown some differences in the intracranial regional ADC changes between male and female fetuses using *in utero* DWI during the second and third trimesters.

## Introduction

A large number of MRI studies have identified numerous sex differences in the anatomy, function, and biochemistry of adult male and female brains (1, 2). These differences may emerge early in brain development during the prenatal period, influenced by gene expression and hormonal factors (3). Moreover, certain developmental processes such as cell proliferation, migration, and differentiation take place exclusively during fetal development. Assessing sex-based structural distinctions in this crucial developmental stage can offer vital mechanistic insights into the apparent structural and functional variations emerging post-birth (4).

The diagnostic value of diffusion-weighted imaging (DWI) in fetal brain imaging has been established for acute conditions, such as hypoxic–ischemic insults, chronic fetal pathologies, and congenital heart diseases, that impact the normal brain development and maturation (5, 6). Apparent diffusion coefficient (ADC) values can be used as a quantitative measure of water diffusion in fetal brain tissues (7). There are complex diffuse changes during different stages of fetal development and brain maturation, reflected in changes in brain water content and myelination (8). Numerous studies (9) have shown normative ADC values across different regions of the fetal brain, underscoring the feasibility of ADC measurement as a reliable and reproducible method for evaluating fetal brain development (10). However, the comprehensive understanding of the normal ADC patterns in early fetal development remains incomplete, particularly regarding sex-specific variations between male and female fetuses. Unraveling these sex-specific variations in ADC measurements can help gain a more comprehensive comprehension of fetal brain growth, offer insights into potential clinical significance, and promote the development of personalized prenatal care strategies.

This study aims to investigate the ADC changes in the intracranial regions using *in utero* DWI and explore the potential variations associated with fetal sex. Furthermore, we will analyze the symmetry of the cerebral hemispheres in the cohort.

## Methods

### Participants

A retrospective study was conducted at our institution, involving the analysis of fetal brain MRI databases from the years 2018 to 2023. These databases were sourced from examinations conducted at our medical center. These data were from pregnant women who were familiar with the procedure and possible risks of the fetal MRI. Prior to the examination, all pregnant participants provided written consent to use their clinical data for research purposes. To confirm the fetuses with normal brain appearance, two pediatric radiologists who had 18 years of experience in fetal MRI assessed all MRI scans.

We established a normative database by scanning low-risk pregnant women enrolled in our cohort. The inclusion criteria are as follows: all singleton pregnancies eligible for fetal brain MRI in our hospital (the limitations of fetal ultrasound were attributed to maternal abdominal wall edema, uterine myomas, oligohydramnios); fetal body malformations without a known association with structural brain abnormalities or heart defects: mild hydronephrosis (11), congenital cystic adenomatoid malformation, intestinal duplication, ovarian cyst, and hepatic cyst; and no fetal brain abnormalities detected in fetal MRI. Fetal age was determined based on the first day of the last normal menstrual period and confirmed by a first-trimester ultrasound scan. Previously, some findings of ADC analyses on this cohort were published to delineate normative data by gestational age (GA) (6). However, those analyses did not explore the potential influence of the fetal sex. Moreover, the sample size and gestational week intervals in the present study cohort differed from those in previous studies.

The exclusion criteria were as follows: twin or multiple pregnancies, fetal CNS or chromosomal abnormalities, fetal abnormalities of non-CNS that may affect CNS development, and perinatal infection. The cases with obvious fetal motion artifacts affecting the determination of fetal sex were excluded from the analysis.

### MRI data acquisition and processing

All fetal brain MRI scans were performed using a Philips 1.5 T MRI scanner with a 16-channel Sense XL Torso Coil. The imaging sequences included steady-state free-precession (SSFP), single-shot turbo spin echo (SSTSE), T1-weighted fast imaging (T1WI), and DWI. DWI sequence was performed in the transverse plane using *b* values of 0 and 700 mm^2^s^−1^. The maximal *b* value of 700 was chosen to increase the signal-to-noise ratio (SNR) of the immature brain for demonstrating optimal contrast in the fetal brain. The following parameters were used: repetition time (TR), 2,494 ms; echo time (TE), 96 ms; slice thickness, 4 mm; field of view (FOV), 280 mm^2^ × 320 mm^2^; matrix, 188 × 125; spacing, 0 mm; flip-angle, 90°. The scan time of the DWI sequence was 60 s. The overall duration of fetal MRI acquisition ranged from 15 to 25 min.

Pregnant women were lying in the supine position or the left side position. Neither the mother nor the fetus took sedatives during the examinations. Firstly, the middle and lower abdomens of pregnant women were scanned in the coronal plane, followed by a focused multiplanar scan of the fetal brain. Subsequently, the fetal chest, abdomen, and pelvis were scanned in the axial, sagittal, and coronal planes, respectively.

### Imaging analysis

**Figure 1:**
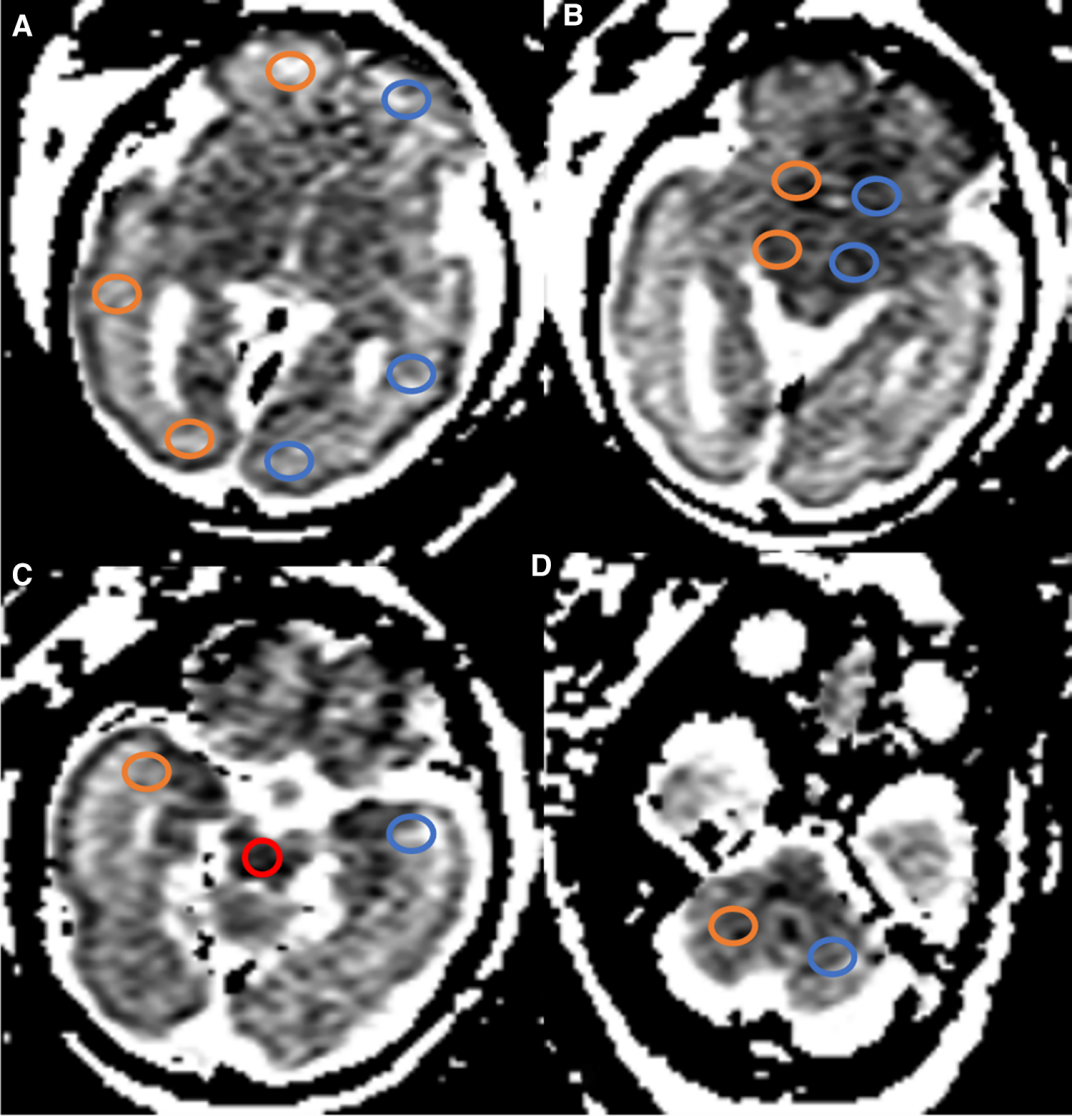
ADC map in a fetus aged 30 weeks showing ROIs in the different regions. (**A–D**) Pairwise ADC values of the ROIs were manually delineated on each side of the FWM, PWM, OWM, BG, THA, TWM, CBM, and a single measurement in the pons (red): orange, right cerebral hemispheres; blue, left cerebral hemispheres. GA, gestational age; ADC, apparent diffusion coefficient; ROIs, regions of interest; FWM, frontal white matter; PWM, parietal white matter; OWM, occipital WM; TWM, temporal WM; BG, basal ganglia; THA, thalamus; CBM, cerebellar hemisphere.

The DWI data were transferred to a workstation (GE HealthCare). ADC measurements were manually delineated in eight circular different regions of interest (ROIs). Pairwise ADC values of the ROIs were manually delineated on either side of the frontal white matter (FWM), parietal WM (PWM), occipital WM (OWM), temporal WM (TWM), basal ganglia (BG), thalamus (THA), cerebellar hemisphere (CBM), and a single measurement in the pons (Figure 1). These ROI placements were based on reported locations in previous literature (12). ROIs varied in size depending on the brain region, and GA ranged from 20 mm^2^ to 60 mm^2^. For each ROI, the mean ADC value (10^−3 ^mm^2^/s) ± standard deviation (SD) from both sides of the fetal brain was averaged for each anatomic location. All ADC measurements were performed manually by the same pediatric neuroradiologist with 5 years of experience in fetal brain MRI. Previous studies have demonstrated good intra- and interobserver reproducibility of this technique (6). Importantly, the pediatric neuroradiologist was blinded to the fetal sex during ADC measurements to mitigate any potential bias.

### Statistical analysis

For each ROI, the mean ADC value was plotted against the GA, and the relationship was assessed by linear regression and polynomial quadratic non-linear analyses for male and female fetuses across various brain regions. The first step of our delineation was to compare ADC values of the labeled hemisphere (right vs. left) by using a paired *t*-test. The relationship of CWM (except for frontal) regions with GA was linear after 28 weeks. Therefore, a general linear model was fitted after this age to assess the rate of decrease of the ADC and to test whether these rates vary by fetal sex. Statistical comparisons between male and female fetuses adjusted for GA were analyzed using the Student's *t*-tests. A *p*-value of <0.05 was considered statistically significant. Correction of false-discovery rates (FDR, *α* = 0.05) was used for multiple testing. Statistical analysis was performed using GraphPad Prism 8.0.0 (GraphPad Software, San Diego) and SPSS 26.0.

## Results

**Figure 2:**
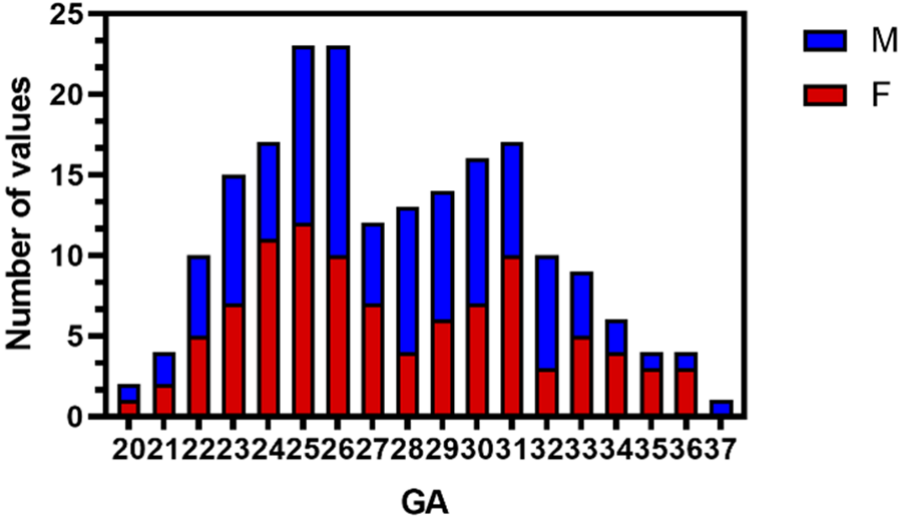
Histogram of GA and sex distribution of MRI scans in normal fetuses (*n* = 200). M, males; F, females; GA, gestational age.

Fetal brain MRI data of 280 singleton pregnancy fetuses were collected. After excluding 80 normal fetal MRI data with noticeable motion artifacts affecting the determination of fetal sex, a total of 100 females (age range, 20–36 weeks; 55 fetuses in 20–27 weeks GA and 45 fetuses in 28–36 weeks GA) and 100 males (age range, 20–37 weeks; 51 fetuses in 20–27 weeks GA and 49 fetuses in 28–37 weeks GA) were analyzed. The mean GA of females was 27.6 ± 4.0 weeks, which was comparable to that of males (27.5 ± 3.7 weeks GA). Figure 2 shows the distribution of fetuses by GA and sex.

**Figure 3:**
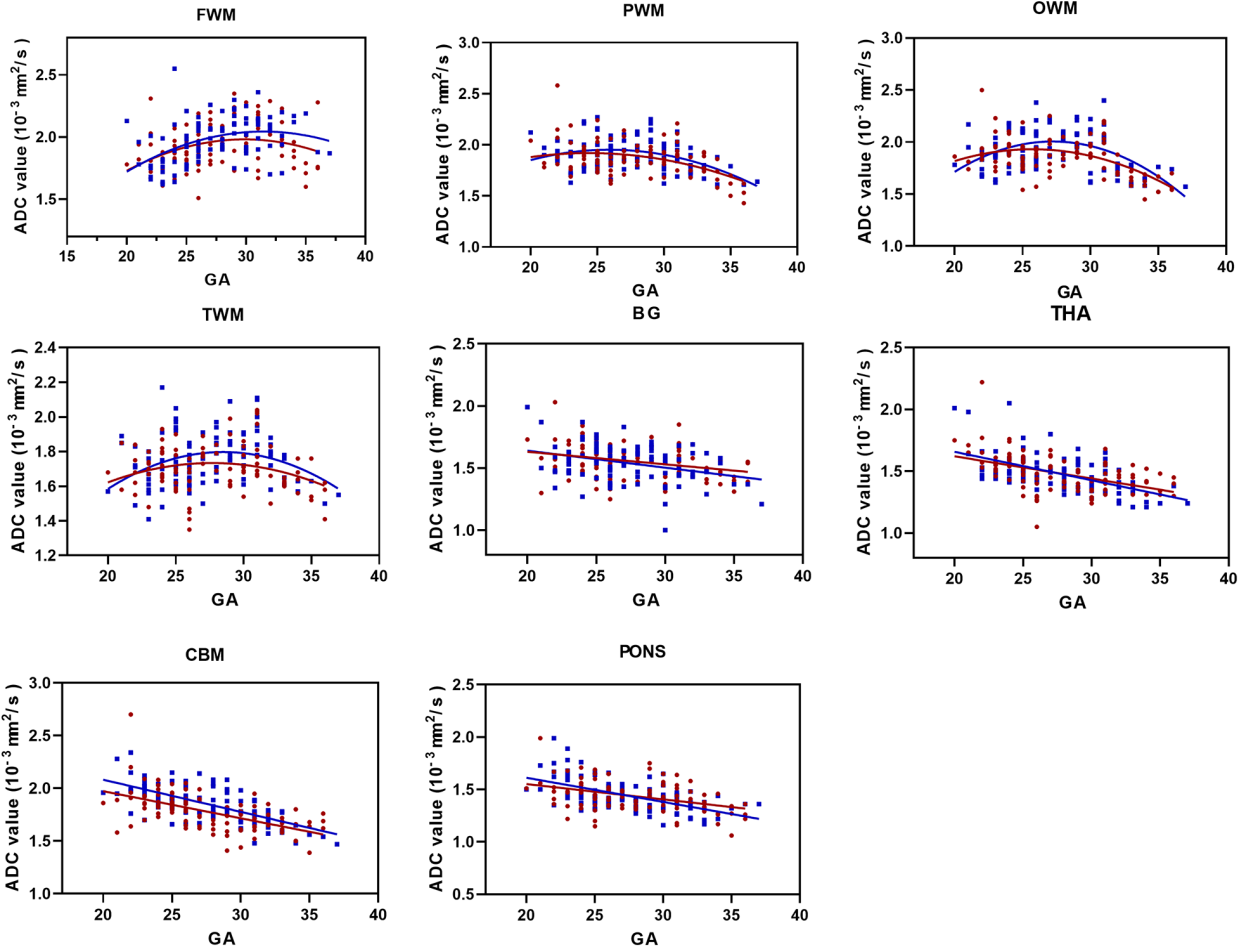
ADC values vs. GA for all ROIs (male fetuses, blue squares; female fetuses, red circles). FWM, PWM, OWM, TWM, BG, THA, CBM, and pons. GA, gestational age; ADC, apparent diffusion coefficient; ROIs, regions of interest; FWM, frontal white matter; PWM, parietal white matter; OWM, occipital WM; TWM, temporal WM; BG, basal ganglia; THA, thalamus; CBM, cerebellar hemisphere.

There was no difference between the sexes in the left and right hemispherical volumes, so the ADC values data were collapsed across hemispheres. For FWM, we found that ADC values were best fit using a quadratic polynomial curve, increasing during the GA of 20–33 weeks and then decreasing afterward until birth. ADC values in the PWM, OWM, and TWM were best fitted using a quadratic polynomial curve, increasing until the GA of 28 weeks and then decreasing progressively afterward (Figure 3). With advancing GA, ADC values in the pons, CBM, THA, and BG all showed strong negative and linear changes (all *p* < 0.05). Table 1 shows the regression slopes against GA and the interaction with sex for fetuses. The main differences in terms of the slopes between male and female fetuses were found for cerebral WM (except for frontal), pons, CBM, THA, and BG with a greater rate decreased in ADC values for males (all *p* < 0.05).

**Table 1:** Regression slopes in relation to gestational age and the interaction with sex for fetuses.

	Female	Male	Difference in slopes	*F*	*p*-value
20 ≤ GA ≤ 37 weeks					
BG	−0.01	−0.014	0.004	11.17	<0.001*
THA	−0.018	−0.023	0.005	4.65	0.01*
CBM	−0.026	−0.030	0.004	63.46	<0.001*
Pons	−0.015	−0.023	0.008	28.54	0.001*
GA ≥ 28 weeks					
FWM	−0.027	−0.003	0.024	2.80	0.07
PWM	−0.04	−0.05	0.01	28.68	<0.001*
OWM	−0.058	−0.059	0.001	33.27	<0.001*
TWM	−0.028	−0.029	0.001	15.51	<0.001*

GA, gestational age; FWM, frontal white matter; PWM, parietal white matter; OWM, occipital WM; TWM, temporal WM; BG, basal ganglia; THA, thalamus; CBM, cerebellar hemisphere.

*Slopes statistically significant.

The *t*-test analysis indicated that the largest sex-related difference was significantly higher ADC values in male fetuses for OWM, TWM, and CBM during the third trimester (all *p* < 0.05). After correction for FDR, the difference in CBM was the only statistically significant (*p =* 0.0032). As for PWM, there was a strong trend between male and female fetuses in late pregnancy, but it did not reach significance (*p* = 0.07) (Table 2).

**Table 2:** ADC values in male and female fetuses in different brain areas.

		Males (*n* = 100)	Females (*n* = 100)	*p*-value
		(Mean ± SD)	(Mean ± SD)	
GA < 28 weeks				
***n***		51	55	
FWM	(10^−3 ^mm^2^/s)	1.92 ± 0.10	1.88 ± 0.6	0.29
PWM	(10^−3 ^mm^2^/s)	1.92 ± 0.03	1.90 ± 0.08	0.41
OWM	(10^−3 ^mm^2^/s)	1.94 ± 0.04	1.90 ± 0.08	0.24
TWM	(10^−3 ^mm^2^/s)	1.72 ± 0.07	1.70 ± 0.05	0.40
BG	(10^−3 ^mm^2^/s)	1.57 ± 0.10	1.54 ± 0.15	0.25
THA	(10^−3 ^mm^2^/s)	1.55 ± 0.06	1.54 ± 0.10	0.67
CBM	(10^−3 ^mm^2^/s)	1.93 ± 0.11	1.87 ± 0.13	0.08
Pons	(10^−3 ^mm^2^/s)	1.48 ± 0.10	1.50 ± 0.13	0.41
GA ≥ 28 weeks				
***n***		49	45	
FWM	(10^−3 ^mm^2^/s)	2.03 ± 0.15	1.98 ± 0.12	0.17
PWM	(10^−3 ^mm^2^/s)	1.89 ± 0.11	1.83 ± 0.03	0.07
OWM	(10^−3 ^mm^2^/s)	1.92 ± 0.11	1.82 ± 0.03	0.02*
TWM	(10^−3 ^mm^2^/s)	1.79 ± 0.11	1.72 ± 0.03	0.01*
BG	(10^−3 ^mm^2^/s)	1.50 ± 0.02	1.52 ± 0.10	0.41
THA	(10^−3 ^mm^2^/s)	1.41 ± 0.11	1.42 ± 0.08	0.89
CBM	(10^−3 ^mm^2^/s)	1.77 ± 0.10	1.66 ± 0.13	0.0002*
Pons	(10^−3 ^mm^2^/s)	1.37 ± 0.06	1.40 ± 0.10	0.14

GA, gestational age; ADC, apparent diffusion coefficient; FWM, frontal white matter; PWM, parietal white matter; OWM, occipital WM; TWM, temporal WM; BG, basal ganglia; THA, thalamus; CBM, cerebellar hemisphere.

* By *t*-test.

**Figure 4:**
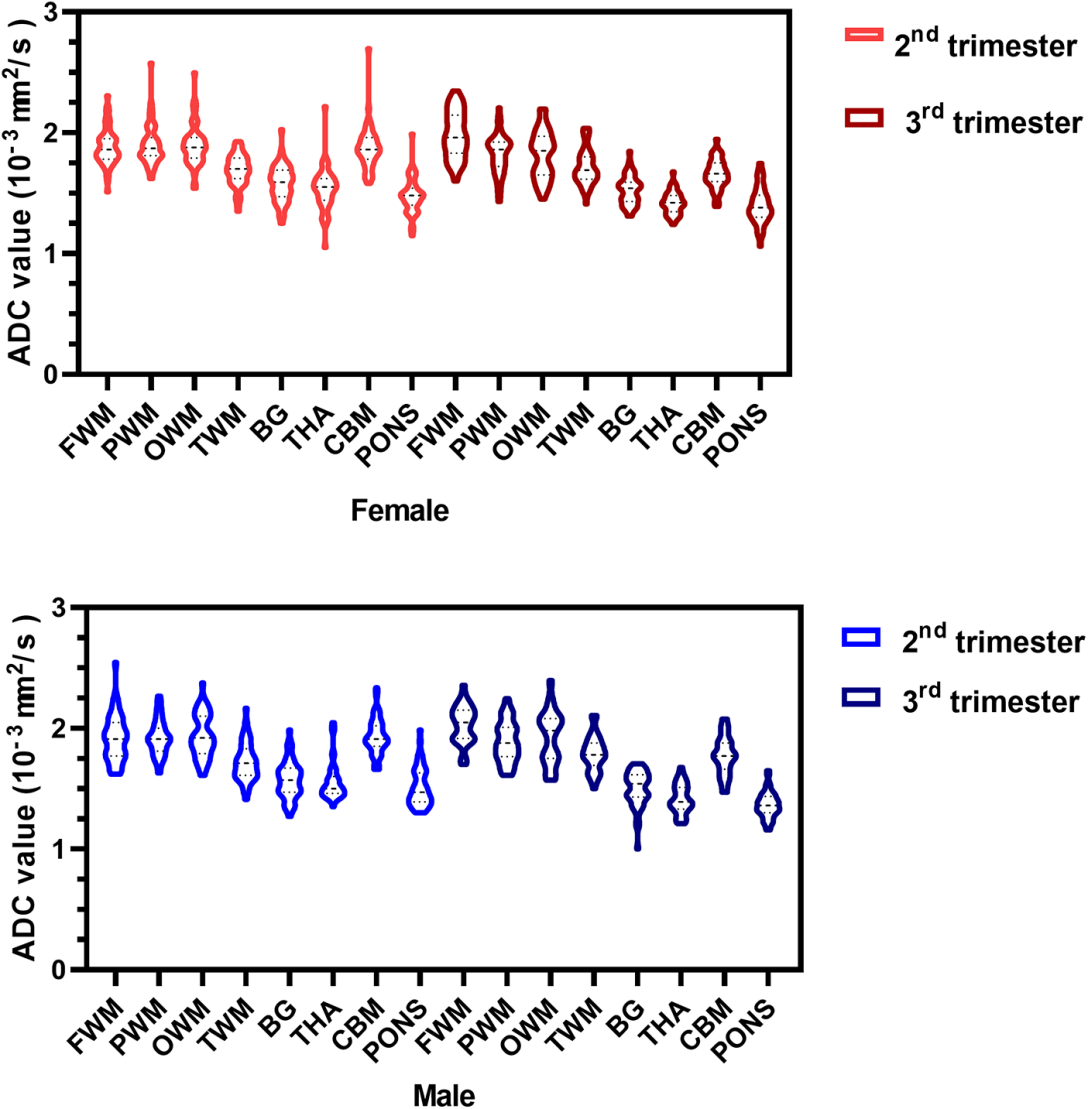
ADC violin plots of the eight representative ROIs in the female (red) and male (blue) fetuses during the second and third trimesters. ADC, apparent diffusion coefficient; ROIs, regions of interest; FWM, frontal white matter; PWM, parietal white matter; OWM, occipital WM; TWM, temporal WM; BG, basal ganglia; THA, thalamus; CBM, cerebellar hemisphere.

Regardless of whether the fetuses were male or female, the mean ADC values of the WM regions (FWM, PWM, OWM) were consistently significantly higher than those of the infratentorial regions (CBM, pons), and the mean ADC values of the deep gray matter (BG, THA) were consistently similar to each other during the second and third trimesters of pregnancy (Figure 4).

## Discussion

In this paper, we have shown the regional differences in ADC values and different rates of decline in ADC values in a large cohort of 200 normal fetuses between 20 and 37 weeks GA. We also found the ADC values in OWM, TWM, and CBM were significantly higher in male fetuses than those in female fetuses during the late stages of pregnancy. After the correction of FDR, the difference in CBM was statistically significant. The decreased rate of the ADC value in male fetuses in BG, THA, CBM, pons, and CWM (except for frontal) ≥28 weeks was higher than that in female fetuses.

Recently, several studies have highlighted substantial differences in fetal head measurements between male and female fetuses, underscoring the prenatal origin of these distinctions. Galjaard et al. (13) used ultrasound to develop sex-specific fetal growth curves, revealing that male fetuses had larger head circumference and biparietal diameter compared to female fetuses. Additionally, Kavak et al. (3) demonstrated the sex-based variations in sizes of the cavum septum pellucidum and anterior horn of the lateral ventricle in the brains of normal fetuses between 20 and 22 weeks GA. Machado-Rivas et al. (14) demonstrated significant sexual dimorphism, with the largest sex-related differences observed in the lateral ventricles, the corpus callosum, and the cerebellar hemispheres. Griffiths et al. (15) used a three-dimensional MRI to assess the total intracranial volume and the brain parenchymal volume increasing at a statistically significantly greater rate in male fetuses than that in female fetuses after 24 weeks GA. Although greater lateral ventricle volumes in male fetuses were evident earlier, the rate of lateral ventricular volume increase was similar for both male and female fetuses.

While ultrasound has traditionally been the preferred imaging modality for prenatal examination, fetal MRI has obvious advantages over ultrasound in displaying neurologic maturation and abnormalities (16). Diffusion MRI provides a unique tool for observing the microstructure and development of fetal brain tissue, potentially aiding in the detection and characterization of diffuse injuries in the fetal brain (17). However, it is noteworthy that studies investigating such sex differences in fetal brain development using DWI with substantial sample sizes are scarce in the current literature. Some technical challenges related to fetal motion, low SNR, and maternal breathing have limited the success of conventional DWI techniques in fetal imaging. In this study, we implemented repeated data collection and/or breath-holding maneuvers at the end of expiration in pregnant women to minimize motion artifacts and enhance the success of *in utero* DWI acquisition.

The development of the brain is related to a variety of factors, including an increase in brain cells, neuronal maturation, and myelination formation (18). Our findings have indicated that the ADC values of the supratentorial WM gradually increased from 20 weeks GA to a peak around 28 weeks GA, followed by a rapid decrease in most brain regions. This pattern may reflect an integrated maturation tissue process, with ADC values reaching a peak around 28 weeks GA (9, 19). Furthermore, our results have revealed that male fetuses exhibited slightly higher ADC values compared to female fetuses in all intracranial regions except for the pons. Consequently, during the early stages of pregnancy (GA < 28 weeks), the higher mean ADC values in the WM of the male fetuses likely indicate earlier maturation. It has been observed that due to the decrease in brain water content with age, the ADC values of the neonatal and fetal brain are higher than those of the adult brain in the same regions, which indicates that the ADC values decrease with age until around 2 years of age in infancy (20). The main differences in terms of the slopes were for cerebral WM (except for frontal) with a significantly greater rate of decrease in ADC values for male fetuses during the later stages of pregnancy. So, the results may indicate that the brain water content of male fetuses decreases faster than that of female fetuses.

While the most significant sex-related difference was a notably higher ADC value in male fetuses for CBM, the decreased rate of ADC values in male fetuses in CWM (except for frontal), BG, THA, CBM, and pons was higher than that in female fetuses during the second and third trimesters. It has also been shown that the process of brain maturation and myelination in normal fetal brains may be from the inside out and from the back to the front (21). Therefore, the ADC values of the regions that mature and myelinate earlier (such as the CBM and pons) are lower than those of the supratentorial WM area. Our study holds significance as it utilizes DWI to investigate the developmental differences in the microstructural organization of the fetal brain between males and females. This approach allows for a more detailed examination of subtle changes in tissue characteristics, offering insights into the underlying mechanisms of sex-specific brain development during the prenatal period. By elucidating these differences, our research contributes to a deeper understanding of neurodevelopmental processes and may have implications for early detection and intervention strategies in developmental disorders.

### Limitations

There are several limitations in this study. Firstly, it was a single-center, retrospective, and cross-sectional study. Due to clinical constraints, no longitudinal follow-up of the fetuses was conducted. However, previous research (16) has indicated a very low false-positive and false-negative rate for detecting fetal brain abnormalities through prenatal MR. Additionally, our study was limited to DWI, and we did not perform diffusion tensor imaging, which has a longer image acquisition time and is more susceptible to fetal motion. Further investigation with a larger sample size is necessary to strengthen the robustness of our conclusions. In addition, we acknowledge the need to incorporate basic experimental research to confirm and extend our findings.

## Conclusion

We have identified some differences in the intracranial regional ADC changes between male and female fetuses using *in utero* DWI during the second and third trimesters. This is the first study to demonstrate the sex differences in the intracranial regional ADC changes with increasing GA *in utero*. We believe that this discovery will provide valuable insights into the assessment of fetal brain development, as ADC is a significant objective indicator of abnormal brain maturation.

## Author's note

The abstract of the article was an oral presentation at the 2023 AOSPR in part.

## Data Availability

The raw data supporting the conclusions of this article will be made available by the authors, without undue reservation.
